# Enterobiasis of the Ovary in a Patient With Cervical Carcinoma In Situ

**DOI:** 10.1155/S1064744995000081

**Published:** 1995

**Authors:** Kevin McCabe, Patricia A. K. Nahn, Aysegul A. Sahin, Michele Follen Mitchell

**Affiliations:** 1 Department of Pathology University of Texas M.D. Anderson Cancer Center Houston TX USA; 2 Department of Gynecologic Oncology University of Texas M.D. Anderson Cancer Center Box 67, 1515 Holcombe Boulevard Houston TX 77030 USA

## Abstract

*Background:* Enterobiasis occurs throughout the female genital tract and may involve peritoneal surfaces. It is generally an incidental finding at surgery or at autopsy but occasionally is symptomatic. Most of the superficial lesions are composed of granulomas with variable fibrosis in which diagnostic eggs are found, often associated with degenerated adult worms. Multiple histologic sections may be required to establish the diagnosis in older lesions.

*Case:* A case of enterobiasis of the ovary in a patient with squamous-cell carcinoma in situ of the cervix is presented. The features of enterobiasis are discussed.

*Conclusion:* The importance of mistaking such lesions for ovarian cancer is discussed.


Infectious Diseases in Obstetrics and Gynecology 2:231–234 (1995)
© 1995 Wiley-Liss, Inc.

### Enterobiasis of the Ovary in a Patient With Cervical Carcinoma In Situ

**Kevin McCabe, Patricia A.K. Nahn, Aysegul A. Sahin, and Michele Follen Mitchell**

*Departments of Pathology (K.M., A.A.S.) and Gynecologic Oncology (P.A.K.N., M.F.M.), University of Texas M.D. Anderson Cancer Center, Houston, TX*

Abstract

***Background:*** **Enterobiasis occurs throughout the female genital tract and may involve peritoneal surfaces. It is generally an incidental finding at surgery or at autopsy but occasionally is symptomatic. Most of the superficial lesions are composed of granulomas with variable fibrosis in which diagnostic eggs are found, often associated with degenerated adult worms. Multiple histologic sections may be required to establish the diagnosis in older lesions.**

***Case:*** **A case of enterobiasis of the ovary in a patient with squamous-cell carcinoma in situ of the cervix is presented. The features of enterobiasis are discussed.**

***Conclusion:*** **The importance of mistaking such lesions for ovarian cancer is discussed.**
© 1995 Wiley-Liss, Inc.

Key words

*Enterobius vermicularis,* pinworm, oxyuriasis, parasitic disease, granulomas

Ovarian and endometrial infection with intraperitoneal dissemination by *Enterobius vermicularis* (pinworm) is a recognized, albeit uncommon, phenomenon. Involvement has been reported in all organs of the female genital tract including the vagina as well as the pelvic peritoneum.[1–4] Granulomas containing the parasite are generally incidental findings in surgical or autopsy tissue specimens in patients without known symptoms. Occasionally, however, the lesions are symptomatic.[5–11] The gross detection of these lesions can be of particular concern in a patient with a malignancy or suspected malignancy because the lesions may mimic metastatic disease.[2] Multiple histologic sections may be required to demonstrate the presence of the organism and hence to identify the etiology of the granulomas. In this report we present such a case.

### CASE REPORT

A 27-year-old woman, $G_5P_4$, who worked in the home was referred to the colposcopy clinic of the University of Texas M.D. Anderson Cancer Center after a routine screening Papanicolaou (Pap) smear revealed the presence of grade III cervical intraepithelial neoplasia (CIN). Her family history was remarkable for a mother with ovarian cancer, a maternal grandmother with ovarian and breast cancer, 2 maternal aunts with breast cancer, and a great maternal aunt with ovarian cancer. Colposcopically directed biopsies showed CIN III with a positive endocervical curettage. A Pap smear confirmed CIN III. She requested definitive therapy. A cone biopsy revealed carcinoma in situ extending to the endocervical margin and a hysterectomy was performed. At the request of the patient, a bilateral salpingo-oophorectomy was also performed in view

**Address correspondence/reprint requests to Dr. Michele Follen Mitchell, Department of Gynecologic Oncology, University of Texas M.D. Anderson Cancer Center, Box 67, 1515 Holcombe Boulevard, Houston, TX 77030.**

Received July 20, 1994
Accepted October 26, 1994

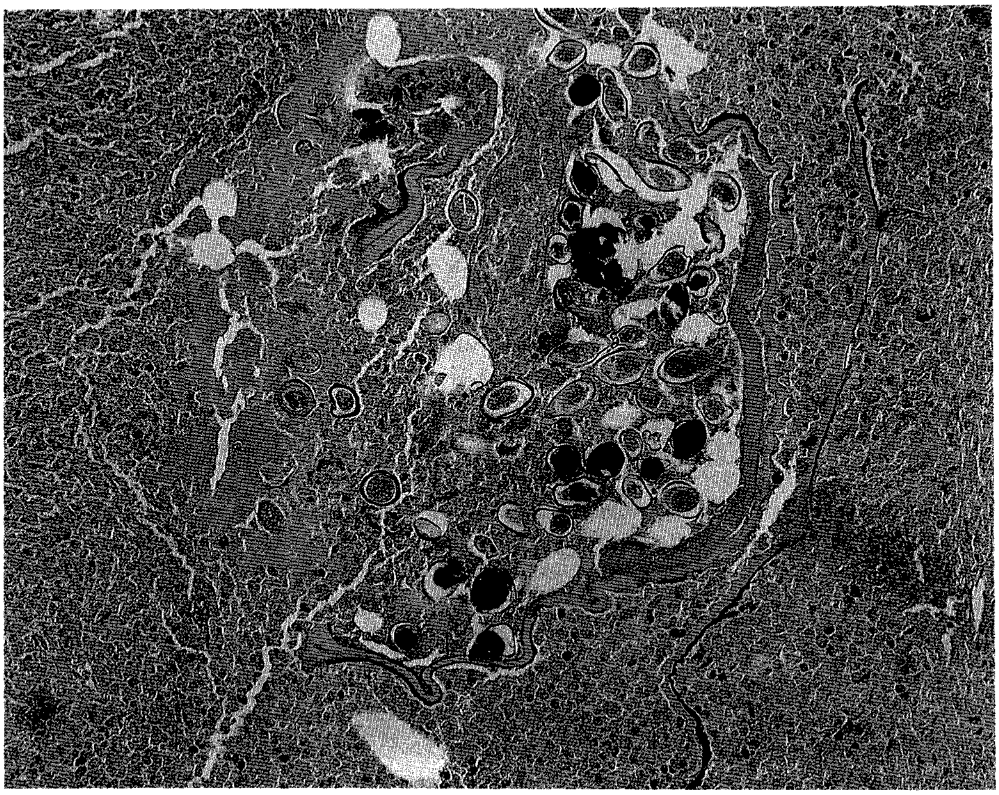

**Fig. 1. A section of the ovary showing fibrotic stroma and granulation tissue. Present in the granulation tissue are eggs with histomorphologic features consistent with *E. vermicularis*.**

of the family history of ovarian neoplasia. The right ovary appeared abnormally nodular. A thorough abdominal inspection revealed no abnormalities. On gross examination, a smooth tan nodule, 2 mm in diameter, was identified on the surface of the right ovary. A slightly smaller nodule was identified on cut section in the ovarian parenchyma near the surface. The ovaries were otherwise grossly unremarkable except for a few small cysts. The uterus was remarkable only for the cone biopsy site; the fallopian tubes appeared normal. Histologically, residual dysplasia was identified in the endocervix, but invasive carcinoma was not identified. The ovarian nodules were composed of granulomas with fibrosis (Fig. 1). Within the granulomas were fragments of degenerated adult parasites and clusters of ovoid eggs, each $50–60 \times 20–30$ μm with double contours and a slightly flattened appearance on one side. Eggs and adult remnants were present in only 2 of 10 sections examined (in only 1 of the 5 sections from each nodule). Although the adult worm remnants were too degenerated for identification, the egg morphology was diagnostic for *E. vermicularis*. No evidence of uterus or fallopian tube involvement was identified histologically. No history of enterobiasis or symptomatology was elicited from the patient when specifically requested. The Medicine and Infectious Disease Services recommended a cellophane-tape test performed 5 times, which revealed no evidence of active parasitic disease. No treatment was prescribed.

### DISCUSSION

This case illustrates many of the typical features of enterobiasis (formerly called oxyuriasis). This case, involving the female genitourinary tract, further shows how the lesions, which may grossly resemble metastatic tumor, can cause concern when found in a patient with an unrelated condition. In this particular case, the ovary itself was the only abnormal-appearing organ. There is one similar report in the literature in which a patient with cervical cancer and a solitary nodule in the ovary was found to have enterobiasis identified histologically.[1]

The lesions in our case were incidental findings in one ovary of a patient who had no detectable active parasitization. Such lesions are the result of an inflammatory response to dead adult female worms that, in most cases, have inadvertently migrated to the peritoneal cavity from the perianal area through the vagina, uterus, or fallopian tubes.[6] This route of peritoneal access accounts for the greater frequency of infection in female patients. Although penetration of the gastrointestinal tract has also been reported as a mechanism of peritoneal involvement, it appears that this route is possible only when there is a preexisting disease that violates the integrity of the bowel of the appendiceal wall. This is the proposed mechanism by which intraperitoneal involvement occurs in males.

Patients with symptomatic disease have been reported to present with clinical features of endometriosis,[8,9] salpingitis,[10] appendicitis,[7] and generalized peritonitis.[5,9] In some cases, the clinical evaluation for symptomatic disease has revealed the presence of a mass in an affected area, such as the ovary,[8] fallopian tube,[10] or vagina.[11] In each of these reports, the mass appeared to be clinically benign.

The lesions of enterobiasis usually consist of superficial granulomas with variable necrosis and fibrosis depending on the age of the lesion. Granulation tissue, acute inflammation, and eosinophils may also be found in early lesions.[2] Fragments of degenerating adult parasites and clusters of eggs from degenerated uteri of gravid worms are identified within the granulomas. The lesions are generally found on the serosal surfaces because the worms typically do not invade normal tissue. However, they have been identified within the parenchyma of otherwise normal ovaries. It has been proposed that parasitization in this location is possible immediately after an ovarian follicle has ruptured and exposed the parenchyma.[1,4]

A diagnosis of enterobiasis is based on the characteristic morphology of the eggs because the adult parasites are usually degenerated. The typical egg is oval with double contours and a slight flattening of one side, measuring 50–60 × 20–30 μm (Fig. 1). As our case illustrates, the diagnostic findings may be very focal; hence, multiple histologic sections may be required to establish the diagnosis.

Once the diagnosis of enterobiasis is established, the patient should be evaluated for active disease which is best accomplished with the cellophane-tape test. The best results are obtained when the tape test is placed either in the evening a few hours before the patient retires to sleep or just after the patient awakes in the morning. One-half of infections are detected with a single test and approximately 99% are detected within 5 samplings.[13] Therefore, the test should be performed at least 5 times on separate days to rule out active disease. A stool examination is generally not rewarding because the eggs are deposited outside the intestinal tract (on the perianal skin) and intact adult worms are usually not passed in the feces. This patient was not acutely infected. Had she been acutely infected, she would have been treated with mebendazole.

### CONCLUSIONS

Enterobiasis may occur throughout the female genital tract and involve peritoneal surfaces. It is generally an incidental finding at surgery or at autopsy and may be confused with a metastatic process. Most of the superficial lesions are composed of granulomas with variable fibrosis in which diagnostic eggs are found, often associated with degenerated adult worms. The diagnosis of older lesions is harder to establish, frequently requiring multiple sections. This case of enterobiasis of the ovary was diagnosed in a patient with squamous-cell carcinoma in situ of the cervix in whom no active disease was found.

### REFERENCES

1. Beckman EN, Holland JB: Ovarian enterobiasis. A proposed pathogenesis. Am J Trop Med Hyg 30(1):74–76, 1981.
2. Fitzgerald TB, Mainwaring AR, Ahmed A: Pelvic peritoneal oxyuriasis simulating metastatic carcinoma. A case report. Br J Obstet Gynaecol 91:248–250, 1974.
3. Garud MA, Saraiya U, Paraskar M, Khohkawalla J: Vaginal parasitosis. Acta Cytol (Baltimore) 24(1):34–35, 1980.
4. Gill AJ, Smith AL: Presence of *Enterobius (oxyuris) vermicularis* in the ovary. Am J Clin Pathol 22:879–882, 1952.
5. Khan JS, Steel RJC, Stewart D: *Enterobius vermicularis* infestation of the female genital tract causing generalized peritonitis. Br J Obstet Gynaecol 88:681–683, 1981.
6. Knuth KR, Fraiz J, Fisch JA, Draper TW: Pinworm infestation of the genital tract. Am Fam Phys 38(5):127–130, 1988.
7. Nutting SA, Murphy F, Inglis FG: Abdominal pain due to *Enterobius vermicularis*. Can J Surg 23(3):286–287, 1980.
8. McMahon JN, Connolly CE, Long SV, Meehan FP: *Enterobius* granulomas of the uterus, ovary and pelvic peritoneum. Two case reports. Br J Obstet Gynaecol 91: 289–290, 1984.

9. Pearson RD, Irons RP Sr, Irons RP Jr: Chronic pelvic peritonitis due to the pinworm *Enterobius vermicularis*. JAMA 245(13):1340–1341, 1981.
10. Saffos RO, Rhatigan RM: Unilateral salpingitis due to *Enterobius vermicularis*. Am J Clin Pathol 57:296–299, 1977.
11. Snow P, Cartwright G, Rumbaugh R: *Enterobius* in an unusual location. JAMA 240(19):2046, 1978.
12. McDonald GSA, Hourihane DO: Ectopic *Enterobius vermicularis*. Gut 13:621–626, 1972.
13. Mandell GL, Douglas RG Jr, Bennett JE (eds): Principles and Practice of Infectious Diseases. 2nd ed. New York: John Wiley & Sons, 1985.
